# Association of GlyCD147 with carotid atherosclerosis: evidence from integrative analyses

**DOI:** 10.1186/s12872-026-05665-4

**Published:** 2026-02-28

**Authors:** Cuihong Tian, Peixuan Yang, Xingang Li, Hongxia Zhang, Jieyi He, Jinxiu Zhu, Yequn Chen, Xuerui Tan

**Affiliations:** 1 Department of Cardiology, First Affiliated Hospital of Shantou University Medical College, Shantou, 515041 Guangdong China; 2 Clinical Medical Research Centre, First Affiliated Hospital of Shantou University Medical College, Shantou, 515041 Guangdong China; 3 Human Phenome Institute of Shantou University Medical College, Guangdong Engineering Research Centre of Human Phenome, Chemistry and Chemical Engineering Guangdong Laboratory, Shantou, Guangdong 515063 China; 4 Glycome Research Institute, Shantou University Medical College, Shantou, 515041 Guangdong China; 5 Molecular Cardiology Laboratory, First Affiliated Hospital of Shantou University Medical College, Shantou, 515041 Guangdong China; 6 Centre for Precision Health, Edith Cowan University, Perth, WA 6027 Australia; 7 Health Care Centre, First Affiliated Hospital of Shantou University Medical College, Shantou, 515041 Guangdong China; 8 Clinical Electrocardiology Institute, First Affiliated Hospital of Shantou University Medical College, Shantou, 515041 Guangdong China; 9 Longgang Maternity and Child Institute of Shantou University Medical College, Shenzhen, 518100 Guangdong China

**Keywords:** Carotid atherosclerosis (CAS), Cluster of differentiation 147 (CD147), Glycosylated CD147 (GlyCD147), Matrix metallopeptidase 9 (MMP9)

## Abstract

**Background:**

Atherosclerosis is an inflammatory cardiovascular disease that progresses with ageing. Glycosylation plays a significant role in inflammation and contributes to the pathogenesis of ageing-related diseases, including atherosclerosis. Cluster of differentiation 147 (CD147), a glycosylated agonist of matrix metallopeptidase 9 (MMP9), serves as an inducer of atherosclerosis and atherothrombosis. However, the effect of glycosylated CD147 (GlyCD147) on atherosclerosis remains unclear. This research aims to identify whether GlyCD147 is associated with atherosclerosis.

**Methods:**

To begin with, a clinical case-control study was designed to assess the differences of CD147 and GlyCD147 between 69 carotid atherosclerosis (CAS) cases and 69 controls. Traditional atherosclerotic risk factors, including age, gender, smoking, alcohol consumption, hypertension, type 2 diabetes mellitus (T2DM), dyslipidemia and obesity, between cases and controls were matched by propensity score matching. Next, a cross-sectional study including 583 participants was conducted to confirm the association of GlyCD147 with CAS by multivariable logistic analysis, which was further examined within subgroups defined by age, gender, smoking, hypertension, T2DM and dyslipidemia. Subsequently, the differentially expressed key genes of carotid artery atheroma were identified by profiling the GSE43292 gene expression dataset.

**Results:**

The serum level of GlyCD147 in CAS cases was higher than that in controls [2.40 µg/L vs. 1.06 µg/L, *P* < 0.001], whereas no significant difference of CD147 protein level was observed. Elevated GlyCD147 was positively associated with risk of CAS (adjusted odds ratio 21.57, 95% confidence interval 13.10-35.52, *P* < 0.001). Especially, GlyCD147 was shown to raise risk of CAS onset across different subgroups. The top hub gene associated with CAS was MMP9 based on its high degree centrality in protein-protein interaction network, which was upregulated in atheroma plaques in comparison to the adjacent tissues.

**Conclusion:**

This research demonstrated that GlyCD147 is independently associated with CAS even with other traditional atherosclerotic risk factors being considered. A potential pathogenesis underlying this association could be that GlyCD147 may be involved in CAS by promoting an MMP9-mediated inflammatory response, a hypothesis that warrants future validation through functional experiments.

**Supplementary Information:**

The online version contains supplementary material available at 10.1186/s12872-026-05665-4.

## Introduction

Atherosclerotic cardiovascular diseases (ASCVD), including ischemic stroke, ischemic heart disease and peripheral arterial disease, is the leading cause of death globally [1]. It has been reported that more than 19 million people died of cardiovascular diseases in 2022, approximately accounting for one-third of all worldwide deaths [2]. Atherosclerosis is the pathogenic basis of ASCVD. Inhibiting and reversing atherosclerotic plaques has typically been the focus in ASCVD research. Ageing, male, smoking, alcohol consumption, hypertension, type 2 diabetes mellitus (T2DM), dyslipidemia and obesity have a cumulative effect on incident atherosclerosis [3]. Recurrent cardiovascular events still occur even when targeting primary, secondary and tertiary prevention of atherosclerosis [4]. This suggests that additional underlying factors to atherosclerosis have yet to be explored.

Cluster of differentiation 147 (CD147), encoded by *BSG* (19p13.3), also known as extracellular matrix metalloproteinase inducer (EMMPRIN) and Basigin (BSG), is a transmembrane inflammatory glycoprotein with two extracellular immunoglobulin domains, and can also be secreted outside. Both the membrane-bound and secreted CD147 induce the secretion of matrix metalloproteinases (MMP9), an enzyme responsible for degrading matrix and regulating the dynamic matrix balance in atherosclerotic plaques [5].

Extensive evidence suggests a possible role of CD147 in the pathogenesis of atherosclerosis [6–8]. Studies have shown that substances causing atherosclerosis, such as low-density lipoprotein (LDL), advanced glycation end products and high glucose, can stimulate the expression of CD147 in inflammatory cells [9–11]. While anti-atherosclerosis drugs, such as atorvastatin, can downregulate the expression of CD147 [12]. Oxidized LDLs (ox-LDLs) can increase the expression and release of soluble CD147, from vascular smooth muscle cells (VSMCs) of human coronary arteries, that can react in autocrine fashion on VSMCs to cause VSMCs to secrete MMP9 [13]. It has also been reported that polymorphisms of the CD147 gene are associated with the formation of carotid atherosclerosis (CAS) [14], a window reflecting the process and degree of systemic atherosclerosis [15, 16], indicating that CD147 is vital in promoting CAS.

Glycosylation, the most important and abundant co- and post-translational modification of protein, is a process that occurs in the Golgi apparatus and endoplasmic reticulum, whereby carbohydrates, also known as glycans, are attached to proteins at specific sites [17, 18]. This modification is essential to maintain the activity and proper function of glycoproteins, including cell-cell recognition, signaling, immune response and disease pathology, by affecting the structure, folding, trafficking, solubility, stability and half-life of proteins [17]. Changes in glycosylation are also associated with inflammatory responses [19] and ageing processes [20]. As a molecule involving in inflammation, CD147 N-glycosylation has been associated with cancers [21, 22] and diabetic myocardium [23]. However, whether glycosylation, one of the most important biological features for CD147, is essential in CAS remains unclear. Based on the above literature, it is hypothesized that glycosylated CD147 (GlyCD147) may be associated with CAS, likely through an MMP9-mediated inflammatory response. This research aims to clarify whether GlyCD147 is associated with CAS.

## Materials and methods

### Study design, setting and participants

To begin with, a clinical case-control study was carried out to assess the differences of serum CD147 and GlyCD147 levels, between 69 CAS patients and 69 controls, by using a propensity score matching (PSM) method, matched 1:1 by sex, age, smoking, alcohol consumption, hypertension, T2DM, dyslipidemia and obesity. Next, a cross-sectional study was applied to validate the association of GlyCD147 with CAS among 583 participants, using multivariate logistic regression analysis and subgroup analysis. All data in the clinical case-control study and the cross-sectional study were extracted from the existing *Health Examination Cohort Study* (HECS, registration number: ChiCTR2100048740). HECS was designed as a single-centre longitudinal prospective cohort study, aiming to explore the association of IgG glycosylation traits with actual clinical cardiovascular events and plaque phenotype. Participants aged from 50 to 65 years and routinely underwent carotid ultrasonography examination, at the Health Care Centre of the First Affiliated Hospital of Shantou University Medical College (SUMC), Guangdong, China, from July 15, 2021 to March 30, 2022, were recruited [24]. The original HECS was approved by the Clinical Ethics Committee of the First Affiliated Hospital of SUMC (No. B-2021-127). Informed consent was obtained from all recruited participants. Given the data in present study were de-identified with low risk to participants, the Human Research Ethics of Edith Cowan University waived the informed consent for the secondary analysis. The study adhered to the principles of the Helsinki Declaration of 1975.

Subsequently, differentially expressed genes (DEGs) of CAS were further investigated. The GSE43292 gene expression dataset, designed as a strictly paired, within-patient, and within-artery study, consisted of 32 carotid atheroma plaque samples (stage IV and over of the Stary classification) and 32 macroscopically intact carotid tissues (stages I and II of the Stary classification) adjacent to the atheroma plaques from 32 patients with hypertension [25]. This dataset was downloaded from Gene Expression Omnibus (GEO) database (http://www.ncbi.nlm.nih.gov/geo), and gene expression arrays were queried using the GPL6244 [HuGene-1_0-st] platform (Affymetrix Human Gene 1.0 ST Array) [25]. DEG screening, Gene Ontology (GO) and Kyoto Encyclopedia of Genes and Genomes (KEGG) enrichment, construction of a protein-protein interaction (PPI) network, and selection of hub genes were applied as a combined approach of the bioinformatic analyses (Additional file).

## Inclusion criteria

Participants were recruited as CAS cases according with the following criteria: diagnosed as having carotid atherosclerotic plaques by carotid ultrasound [26], *Han* Chinese, and age from 50 to 65 years old. Participants with the following criteria were considered as controls: *Han* Chinese, age from 50 to 65 years old, and without carotid atherosclerotic plaques based on carotid ultrasound examination. Carotid arteries of all participants were bilaterally scanned using ultrasonography. Abnormal carotid intima media thickness (CIMT) was defined as a thickness of 0.9 mm or more [27]. A carotid plaque was defined as a focal CIMT of 1.5 mm or more encroaching into the lumen or at least 0.5 mm or 50% compared with the surrounding CIMT values [28]. The existence of carotid plaques was regarded as CAS in this study [29].

## Exclusion criteria

Participants were excluded based on the following criteria: complicated with ischemic stroke, ischemic heart disease, peripheral arterial disease, diabetes, infection, immune system disease, hematologic system disease or tumor; currently undergoing or having a history of lipid-lowering, anticoagulant, antiplatelet or vasodilator treatment; and incomplete, repeated, or dubious data.

## Measures

Serum levels of CD147 protein and GlyCD147 were examined by a human CD147 enzyme-linked immune sorbent assay (ELISA) kit (SenBeiJia, Nanjing, China) and a human GlyCD147 ELISA kit (Mlbio, Shanghai, China), respectively. Demographic information: sex and age; lifestyles: smoking status and alcohol consumption; medical histories: hypertension (systolic blood pressure ≥ 140 mmHg and/or diastolic blood pressure ≥ 90 mmHg, or use of antihypertensive drugs, or self-reported history of physician-diagnosed hypertension [30]), T2DM (fasting blood glucose ≥ 7.0 mmol/L, or glycohemoglobin A1c levels > 6.5%, or self-reported use of anti-diabetic medication, or self-report of a physician diagnosis [31]), dyslipidemia (total cholesterol ≥ 6.22 mmol/L, or triglyceride ≥ 2.26 mmol/L, or high-density lipoprotein cholesterol < 1.04 mmol/L, or low-density lipoprotein cholesterol ≥ 4.14 mmol/L [32]) and obesity [body mass index ≥ 28 kg/m^2^, calculated as weight (kg) divided by height squared (m^2^)] [33], were extracted from existing datasets within the HECS.

### Blood sample collection

Fasting blood samples (3-4mL) were collected from the antecubital vein of all participants between 8:00am and 9:00am using 5mL vacuum serum separation tubes without anticoagulant. Samples were allowed to clot at room temperature (20–25℃) for 30 min, then centrifuged at 4℃ and 3000 rpm for 10 min. The serum supernatant was carefully aspirated, aliquoted into sterile EP tubes (200 µL per tube, with 2–3 tubes per sample), and stored at -80℃ within 30 min after aliquoting. Samples were stored for 2–6 months, avoiding repeated freeze-thaw cycles. All ELISA measurements were performed in the same batch according to the kit instructions to minimize inter-assay variability.

### Statistical analysis

In the clinical case-control study, PSM was applied using logistic regression to match traditional atherosclerosis-related risk factors, including gender, age, smoking history, alcohol consumption history, hypertension, T2DM, dyslipidemia, and obesity. The matching strategy was 1:1 without replacement, and a caliper of 0.2 times the standard deviation of the propensity score was set to control for matching bias. The standardized mean difference (SMD) values of all covariates were calculated, and the propensity score kernel density plots before and after matching were visualized.

After PSM, the differences of quantitative variables between paired participants were tested for normality. Variables with differences following a normal distribution were expressed as mean ± standard deviation (SD), and the two-tailed paired sample *t-*test was used for pair comparison. Variables with differences following non-normal distribution were described by the median and interquartile range [P25, P75], and the Wilcoxon signed-rank test was used to compare their differences. Categorical variables were described by number and percentage (%) and compared via a McNemar’s test (paired *chi*-squared test).

In the case-control study before PSM and the cross-sectional study, the normality of the quantitative data distribution was checked by the *Kolmogorov-Smirnov* test. Two sample *t* test and Mann-Whitney *U* test were used to compare the difference of variables with normal and skewed distributions, respectively. The *chi*-square test was used to compare the difference of categorical variables. The cut-off value of GlyCD147 to determine patient groupings was calculated by receiver operating characteristic (ROC) curve analysis. Multivariate logistic regression was performed to evaluate the association of GlyCD147 with CAS. Subgroup analysis was conducted to evaluate the association of GlyCD147 with CAS in the stratified participant groups and the interactions of GlyCD147 with other covariates. Estimated odds ratios (OR) and 95% confidence intervals (95% CI) were reported. Statistical analysis was conducted by using SPSS (version 28.0) and R (version 4.2.2) software. *P* < 0.05 was considered to indicate statistical significance.

## Results

### Basic characteristics and serum levels of CD147 and GlyCD147 in CAS patients and controls

**Fig. 1:**
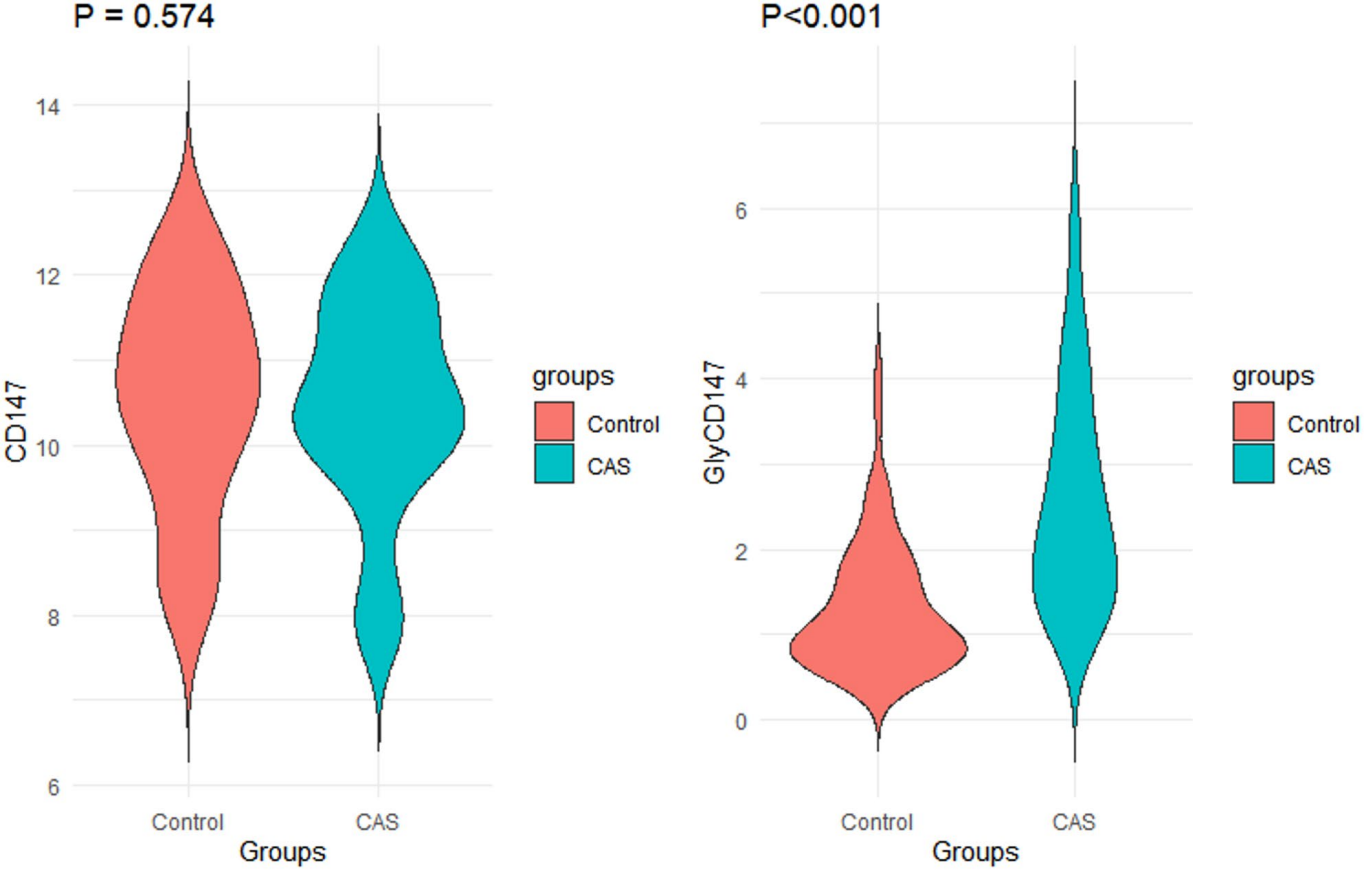
Differential serum levels of CD147 and GlyCD147 in the clinical case-control study. The concnetration unit of CD147 and GlyCD147 is µg/L. Abbreviations: CAS: carotid atherosclerosis; CD147: cluster of differentiation 147

Before PSM, the SMD values of core confounding factors such as age, smoking history, alcohol consumption history, T2DM, and dyslipidemia were all greater than 0.2, indicating severe baseline imbalance between the two groups; after PSM, the SMD values of all confounding factors were less than 0.2, among which the SMD values of gender, smoking history, alcohol consumption history, hypertension, T2DM, and obesity were less than 0.1, meeting the statistically recognized balance standard (Table S1). From propensity score kernel density plots, it can be seen that the propensity score curves of two groups were significantly separated before PSM, reflecting significant baseline imbalance; after PSM, the two curves overlapped highly, visually demonstrating that PSM effectively balanced the confounding factors between the two groups (Fig. S1). After PSM, there was no statistical difference regarding serum CD147 protein level between CAS patients and controls (*P* = 0.574). However, the serum level of GlyCD147 in CAS patients was higher than that in controls [2.40 (1.48, 3.96) µg/L vs. 1.06 (0.76, 1.78) µg/L, *P* < 0.001] (Fig. 1).

### Participant grouping for the cross-sectional study

**Fig. 2:**
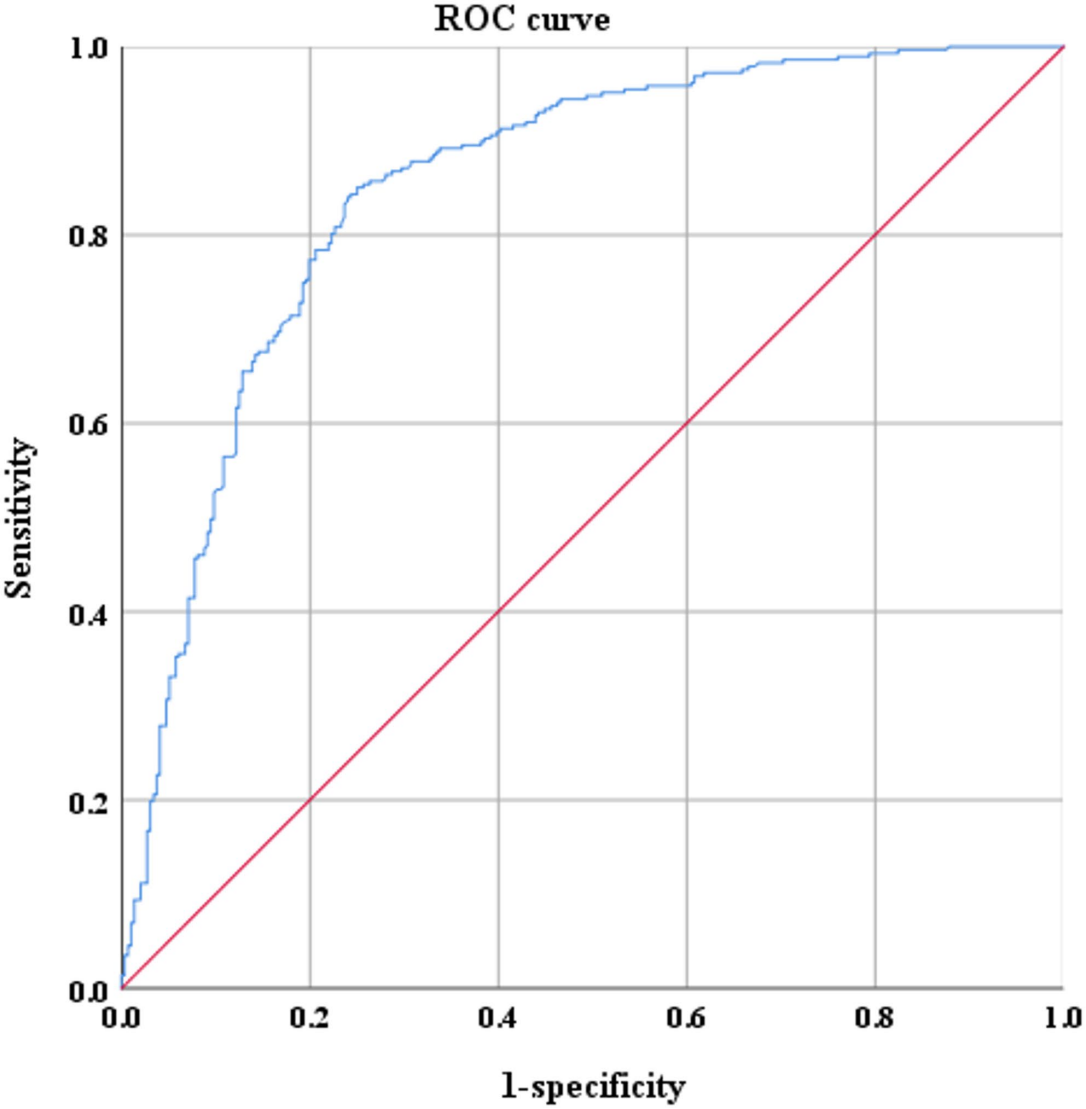
ROC curve analysis for grouping. The AUC was 0.85 (95% CI 0.82–0.88, *P* < 0.001), the cut-off was 1.81 µg/L, sensitivity was 0.84, and specificity was 0.76. Abbreviations: ROC: receiver operating characteristic

In the cross-sectional study, the optimal cut-off value of GlyCD147 to diagnose CAS was 1.81 µg/L, based on a sensitivity of 84%, a specificity of 76% and an area under the curves (AUC) of 85% (Fig. 2). The participants were then divided into a low GlyCD147 group (GlyCD147 < 1.81 µg/L, *n* = 271) and a high GlyCD147 group (GlyCD147 ≥ 1.81 µg/L, *n* = 312) according to the optimal GlyCD147 cut-off value.

### Basic characteristics of participants in the cross-sectional study

Table 1 shows the baseline characteristics of participants in the cross-sectional study. The proportion of females in the low GlyCD147 group was greater than that in the high GlyCD147 group (46.5% vs. 38.1%, *P* = 0.042). Participants in the high GlyCD147 group were older than those in the low GlyCD147 group (57.54 ± 4.40 vs. 56.04 ± 4.58, *P* < 0.001). Participants in the high ClyCD147 group were more likely to smoke (18.6% vs. 9.6%, *P* = 0.002) and develop CAS (77.2% vs. 17.0%, *P* < 0.001), compared with the low GlyCD147 group. The serum level of CD147 in the low GlyCD147 group was higher than that in the high GlyCD147 group [10.12(9.34, 10.85) µg/L vs. 9.56 (8.72, 10.50 µg/L), *P* < 0.001]. The percentage of alcohol consumption, hypertension, T2DM, dyslipidemia and obesity were not significantly different between the two groups (*P* > 0.05).

**Table 1:** Basic characteristics of participants in the cross-sectional study

Variables	Total (*N* = 583)	GlyCD147 < 1.81 (*n* = 271)	GlyCD147 ≥ 1.81 (*n* = 312)	*P*-value
Female, %	245 (42.0)	126 (46.5)	119 (38.1)	0.042
Age, y	56.84 ± 4.57	56.04 ± 4.58	57.54 ± 4.40	< 0.001
Smoking, %	84 (14.4)	26 (9.6)	58 (18.6)	0.002
Alcohol consumption, %	28 (4.8)	13 (4.8)	15 (4.8)	0.995
Hypertension, %	111 (19.0)	44 (16.2)	67 (21.5)	0.108
T2DM, %	90 (15.4)	36 (13.3)	54 (17.3)	0.180
Dyslipidemia, %	258 (44.3)	115 (42.4)	143 (45.8)	0.410
Obesity, %	28 (4.8)	13 (4.8)	15 (4.8)	0.988
BMI, kg/m^2^	23.58 (23.28, 24.90)	23.58 (23.15, 24.80)	23.58 (23.40, 24.95)	0.681
TG, mmol/L	1.38 (1.02, 1.89)	1.36 (0.97, 1.86)	1.41 (1.05, 1.94)	0.582
TC, mmol/L	5.46 ± 1.11	5.42 ± 1.09	5.50 ± 1.13	0.363
HDL-C, mmol/L	1.37 ± 0.36	1.40 ± 0.37	1.35 ± 0.34	0.070
LDL-C, mmol/L	3.34 ± 0.94	3.36 ± 0.85	3.33 ± 1.01	0.703
FBG, mmol/L	5.76 (5.40, 6.36)	5.73 (5.41, 6.23)	5.81 (5.38, 6.48)	0.593
SBP, mmHg	132 (125, 137)	132 (123, 135)	132 (127, 138)	0.012
DBP, mmHg	84 (80, 88)	84 (79, 88)	84 (80, 88)	0.380
CD147, µg/L	9.87 (8.98, 10.68)	10.12(9.34, 10.85)	9.56 (8.72, 10.50)	< 0.001
CAS, %	287 (49.2)	46 (17.0)	241 (77.2)	< 0.001

*Abbreviations BMI* body mass index, *CAS* carotid atherosclerosis, *CD147* cluster of differentiation 147, *DBP* diastolic blood pressure, *FBG* fasting blood glucose, *GlyCD147* glycosylated CD147, *HDL-C* high-density lipoprotein cholesterol, *LDL-C* low-density lipoprotein cholesterol, *SBP* systolic blood pressure, *TC* total cholesterol, *TG* triglyceride, *T2DM* type 2 diabetes mellitus

### Association of GlyCD147 and CAS

**Fig. 3:**
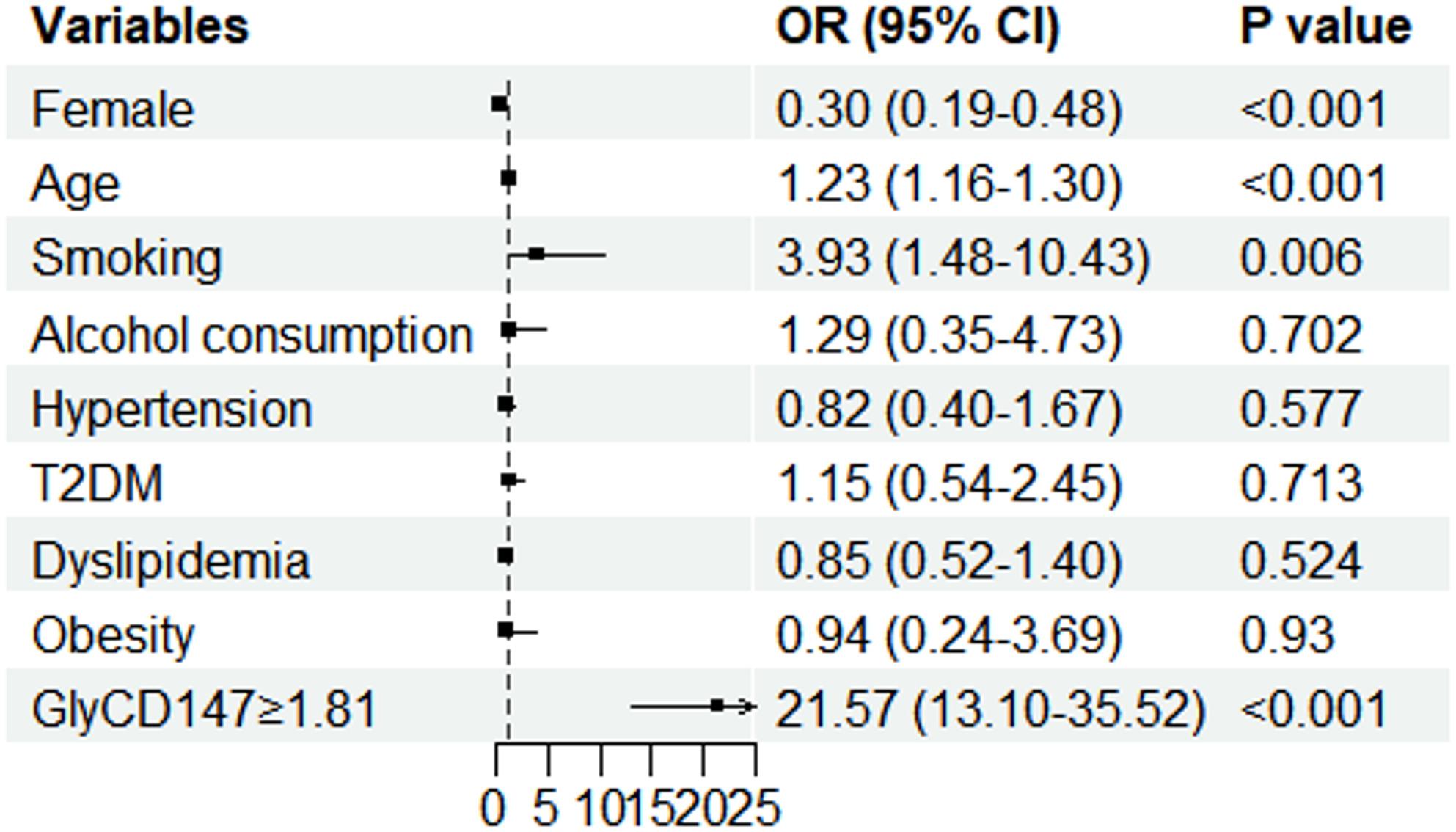
Multivariable analyses of the factors associated with CAS in the cross-sectional study. Age, gender, smoking, alcohol consumption, hypertension, T2DM, dyslipidaemia, and obesity were adjusted. Abbreviations: CD147: 147 cluster of differentiation; GlyCD147: glycosylated CD147; 95% CI: 95% confidence intervals; T2DM: type 2 diabetes mellitus; OR: odds ratios

Univariable analyses were applied to screen the potential risk factors for CAS (Table 2). Multivariable logistic regression analyses were used to evaluate the association between GlyCD147 and CAS, adjusting for variables with a *P* < 0.1 in univariate analysis (i.e., female, age, smoking history, alcohol consumption history, hypertension, and T2DM) that decreased the risk of missing potential confounding factors and a priori predefined CAS-related risk factors with a *P >* 0.1 in univariate analysis (i.e., dyslipidemia and obesity). Age (OR 1.23, 95%CI 1.16–1.30, *P* < 0.001), smoking (OR 3.93, 95%CI 1.48–10.43, *P* = 0.006) and high GlyCD147 (OR 21.57, 95%CI 13.10-35.52, *P* < 0.001) were positively associated with CAS, whereas being female was negatively associated with CAS (OR 0.30, 95%CI 0.19–0.48, *P* < 0.001) (Fig. 3). Each one-standard-deviation increase in GlyCD147 was independently associated with an increased risk of CAS (adjusted OR = 8.84, 95%CI 5.39–14.52, *P* < 0.001) after adjustment for the above covariates.

**Table 2:** Univariable analysis of the factors associated with CAS in the cross-sectional study

Variables	CAS (*n* = 287)	Controls (*n* = 296)	OR	95%CI	*P*-value
Female, %	88 (30.7)	157 (53.0)	0.392	0.279–0.550	< 0.001
Age, y	58.49 ± 4.32	55.24 ± 4.22	1.185	1.138–1.234	< 0.001
Smoking, %	65 (77.4)	19 (22.6)	4.269	2.486–7.331	< 0.001
Alcohol consumption, %	19 (6.6)	9 (3.0)	2.261	1.005–5.084	0.049
Hypertension, %	66 (23.0)	45 (15.2)	1.666	1.095–2.535	0.017
T2DM, %	57 (19.9)	33 (11.1)	1.975	1.242–3.141	0.004
Dyslipidemia, %	136 (47.4)	122 (41.2)	1.285	0.926–1.782	0.134
Obesity, %	18 (6.3)	10 (3.4)	1.921	0.871–4.236	0.106
CD147, µg/L	9.78 (8.59, 10.47)	9.96 (9.25, 10.84)	1.072	0.980–1.173	0.130
GlyCD147, µg/L	2.57 (1.98, 3.64)	1.36 (0.94, 1.78)	2.942	2.360–3.668	< 0.001
GlyCD147 ≥ 1.81, %	241 (84.0)	71 (24.0)	16.603	10.985–25.094	< 0.001

*Abbreviations* *CAS* carotid atherosclerosis, *CD147* cluster of differentiation 147, *GlyCD147* glycosylated CD147, *95% CI* 95% confidence intervals, *OR* odds ratios, *T2DM* type 2 diabetes mellitus

### Subgroup analysis of the association between GlyCD147 and CAS

**Fig. 4:**
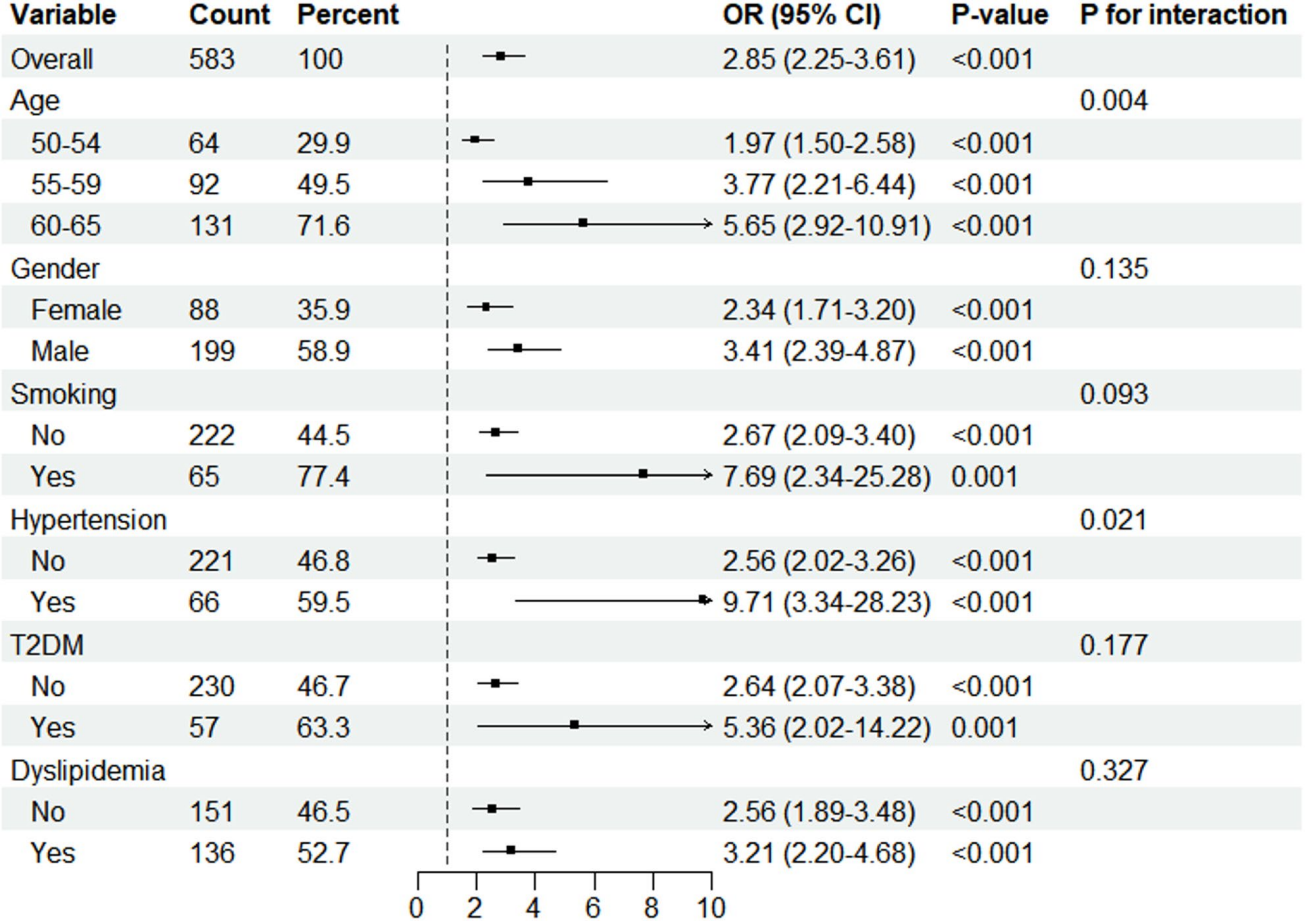
Subgroup analysis of the association between GlyCD147 and CAS. Age, gender, smoking, alcohol consumption, hypertension, T2DM, dyslipidaemia, and obesity were adjusted in this subgroup analysis

Subgroup analysis of the association between GlyCD147 and CAS showed that GlyCD147, analyzed as a quantitative measure, was correlated with an increased risk of CAS across stratified participant groups defined by age, gender, smoking, hypertension, T2DM and dyslipidemia **(**Fig. 4**)**. Significant interactions of GlyCD147 with age and hypertension were identified (*P* for interaction < 0.05). The association of GlyCD147 with CAS was more prominent in older participants than relatively young participants [OR 5.65 (95%CI 2.92–10.91) for participants 60–65 years vs. OR 3.77 (95%CI 2.21–6.44) for those 55–59 years vs. OR 1.97 (95%CI 1.50–2.58) for those 50–54 years], and in participants with hypertension than without hypertension [OR 9.71 (95%CI 3.34–28.23) vs. OR 2.56 (95%CI 2.02–3.26)]. Although no significant interactions were observed between GlyCD147 and gender, smoking, T2DM and dyslipidemia, the association of GlyCD147 with risk of CAS was more distinctive between males and females [OR 3.41 (95%CI 2.39–4.87) vs. OR 2.34 (95%CI 1.71–3.20], between smokers and non-smokers [OR 7.69 (95%CI 2.34–25.28 vs. OR 2.67 (95%CI 2.09–3.40)], between participants with T2DM and without T2DM [OR 5.36 (95%CI 2.02–14.22 vs. OR 2.64 (95%CI 2.07–3.38)], and between participants with dyslipidemia and without dyslipidemia [OR 3.21 (95%CI 2.20–4.68) vs. OR 2.56 (95%CI 1.89–3.48)].

### Identification of DEGs in CAS

To identify DEGs between carotid plaques and adjacent tissues, GSE43292 was acquired from GEO datasets after standardizing microarray results. When setting up a standard of |log_2_FC|>1 and adjusted *P* < 0.05, 147 DEGs overlapped between carotid plaques and adjacent tissues, with 86 upregulated DEGs and 61 downregulated DEGs in carotid plaques identified (Fig. S2a, b). The top ten upregulated and downregulated DEGs of GSE43292 are shown in Table S2.

### GO and KEGG pathway enrichment analyses of DEGs

The database tool for annotation, visualization and integrated discovery (DAVID) was used to analyze the biological function and explore the most promising pathways of DEGs. GO enrichment analysis revealed that DEGs in biological processes (BP), cellular component (CC) and molecular function (MF) were considerably enriched in immune response, plasma membrane and calcium ion binding, respectively. KEGG enrichment analysis showed that DEGs in GSE43292 were significantly enriched in the cyclic adenosine monophosphate (cAMP) signaling pathway (Table S3).

### Construction of a PPI network and selection of hub genes

**Fig. 5:**
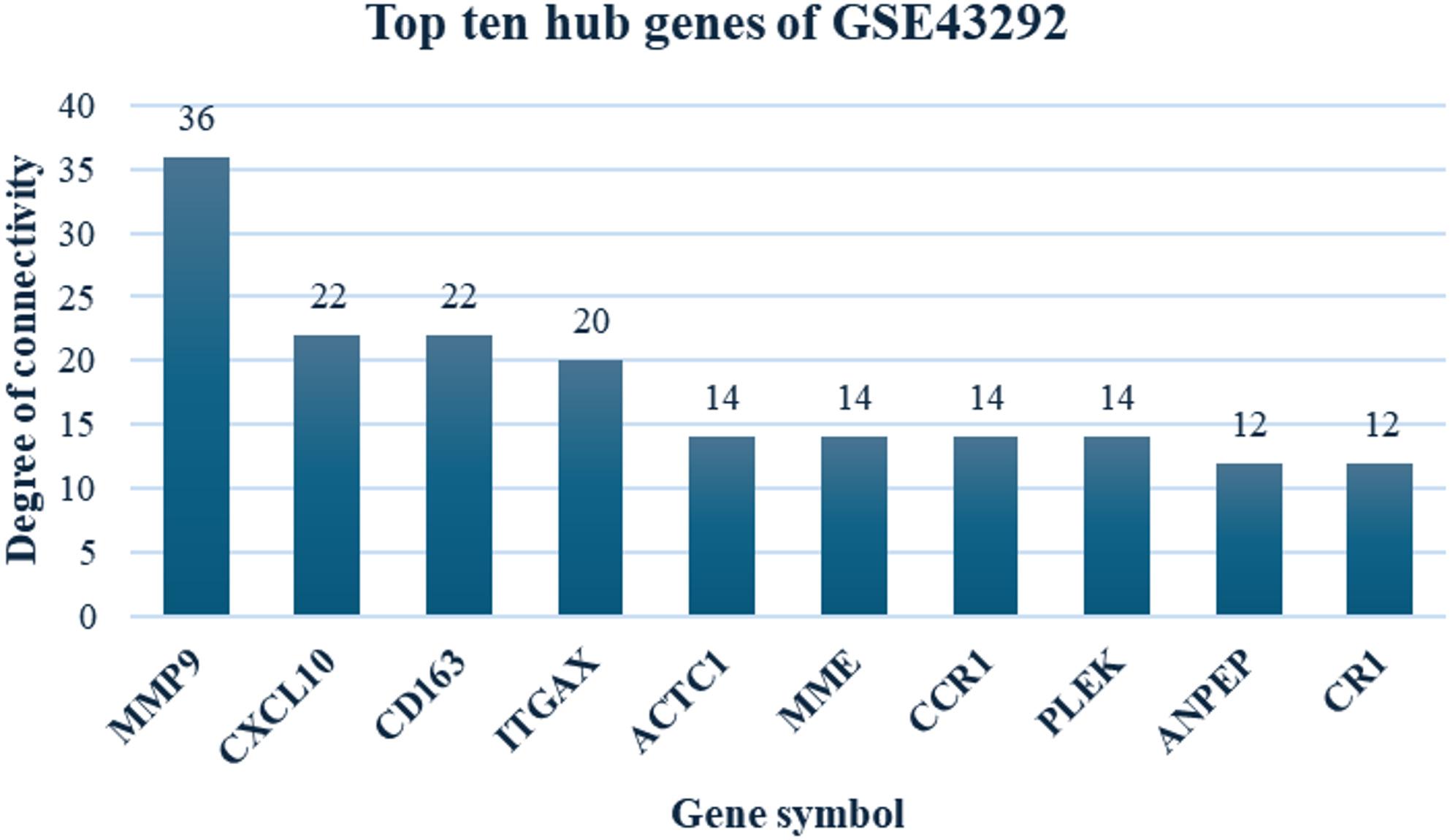
Top ten hub genes of GSE43292 with high degree of connectivity. Abbreviations: *ACTC1*: actin, alpha, cardiac muscle 1; *ANPEP*: alanyl aminopeptidase, membrane; *CCR1*: C-C motif chemokine receptor 1; *CD163*: cluster of differentiation 163; *CR1*: complement component 3b/4b receptor 1 (Knops blood group); *CXCL10*: C-X-C motif chemokine ligand 10; *MME*: membrane metallo-endopeptidase; *MMP9*: matrix metallopeptidase 9; *PLEK*: pleckstrin; *ITGAX*: integrin subunit alpha X

A PPI network of the DEGs in GSE43292, employed by the search tool for retrieval of interacting genes (STRING), is presented in Fig. S3, with 94 nodes and 110 edges. The cytoHubba plugin was employed to screen the top ten hub genes in GSE43292 according to connectivity degree. The results indicated MMP9 to be the most outstanding gene, with a connectivity degree equaling 36, followed by integrin subunit C-X-C motif chemokine ligand 10 (CXCL10, degree = 22), cluster of differentiation 163 (CD163, degree = 22), integrin subunit alpha X (ITGAX, degree = 20), actin, alpha, cardiac muscle 1 (ACTC1, degree = 14), membrane metallo-endopeptidase (MME, degree = 14), C-C motif chemokine receptor 1 (CCR1, degree = 14), pleckstrin (PLEK, degree = 14), alanyl aminopeptidase, membrane (ANPEP, degree = 12) and complement component 3b/4b receptor 1 (CR1, degree = 12) (Fig. 5). Most of these hub genes, except for ACTC1, were upregulated in carotid atheroma.

## Discussion

The present study, conducted from three different perspectives, including a case-control study, a cross-sectional study, and a bioinformatic analysis, demonstrated that GlyCD147 is independently associated with CAS even when considering traditional atherosclerotic risk factors, supporting a potential role of GlyCD147 in CAS, possibly via an MMP9-mediated inflammatory response. The clinical case-control study implied that GlyCD147, the functional form of CD147, is highly expressed in CAS. This finding was further confirmed by the subsequent cross-sectional study, which demonstrated the significant association of GlyCD147 with CAS. Bioinformatic analyses identified MMP9 as the top hub gene in CAS, providing a molecular basis for studying GlyCD147, a well-documented agonist of MMP9, and supporting the proposed mechanism.

The present case-control study revealed that GlyCD147 but not total CD147 elevated in CAS by matching covariates using PSM. Our cross-sectional study further confirmed the independent association between GlyCD147 and CAS by adjusting for traditional atherosclerotic risk factors using multivariable binary logistic analysis. Previous studies investigated the role of CD147 in sparking atherosclerosis by targeting inflammation but paid less attention on its functional form, i.e., GlyCD147 [34]. Actually, glycosylation regulates the size of atherosclerotic plaque via inflammatory response. It has been reported that knockdown of glycosyltransferase genes, such as core 2 β-1, 6-N-acetylglucosaminyltransferase-1 (GCNT1) and α-2, 3-sialyltransferase IV (ST3gal-IV), considerably reduce the size of atherosclerotic lesions by suppressing inflammatory leukocyte recruitment in apolipoprotein E-deficient mice [35, 36]. Additionally, glucosamine and thiamet-G, two medicines targeting glycosylation, exert a protective effect on ischemic stroke via inhibiting the inflammatory response [37, 38]. The present findings precisely proved that the post-translational modification of CD147, i.e., glycosylation, is the functional form of CD147 modulating plaque and attenuating plaque inflammation.

Our bioinformatic analysis identified that MMP9, a member of matrix metalloproteinase (MMP) family, is the top hub gene, and is upregulated in carotid atheroma compared with adjacent intact carotid tissues. Metabolism, immunity, and inflammation collectively shape the local immune responses and the cellular microenvironment of atherosclerotic plaques [39, 40]. MMP9 plays a crucial role in extracellular matrix remodeling, tissue repair, inflammation, and immunity by degrading the matrix and regulating matrix dynamic balance [41]. Further, it involves various pathological conditions, including atherosclerosis [42], viral myocarditis [43], cancer [44], brain injury [45, 46], diabetic nephropathy [47] and inflammatory bowel diseases [48]. It has been reported that MMP9 is associated with an unstable plaque phenotype, leading to thrombotic events and cardiovascular complications [49]. CD147, as an upstream agonist of MMP9, has been shown to activate the expression of MMP9 [50]. Notably, CD147 can be glycosylated to different degrees, forming lowly glycosylated (LG, 32-44 kDa) and highly glycosylated (HG, 45-65 kDa) forms, which mediate the development of non-infectious inflammation and the activation of MMPs, respectively [51]. In comparison, deglycosylated CD147 (27 kDa) can prevent the induction of MMPs in *vitro* [52]. The above evidence, combined with the findings of the bioinformatic analysis, supports a plausible hypothesis that GlyCD147 may be associated with the formation and progression of CAS by inducing the expression of MMP9, which in turn promotes extracellular matrix remodeling, triggers inflammation, and exacerbates plaque instability.

Consistent with previous studies, subgroup analysis in the cross-sectional study showed significant interactions of age and hypertension with GlyCD147 in assessing the risk factors of CAS, indicating that they work together to contribute to CAS. Endothelial lesions of atherosclerosis begin in childhood and progress gradually with age [53]. The Bogalusa Heart Study reveals that the incidence of coronary lipid streaks increased from 50% in the ages between 2 and 15 years to 85% between the ages of 21–39 years, and the incidence of fibrous plaques increases from 8% in the ages of 2–15 to 69% in the ages of 26–39 [54]. Although the GSE43292 dataset is derived from patients with hypertension, the matching of hypertension in the case-control study, the adjustment of hypertension in the cross-sectional study, and the subgroup analysis defined by hypertension jointly revealed that hypertension may not confound CAS-related transcriptomic differences identified in the GSE43292 dataset, but instead potentiate the effect of GlyCD147 on carotid atherosclerotic lesion progression. Specifically, elevated blood pressure exerts mechanical stress on vascular endothelial cells, disrupts endothelial barrier function, and increases permeability to lipids and inflammatory cells. This pro-atherosclerotic microenvironment amplifies the pathogenic effects of GlyCD147, thereby accelerating the development and progression of atherosclerosis [55, 56].

Although no interactions of gender, smoking, T2DM and dyslipidemia with GlyCD147 were observed in our subgroup analysis, the association of GlyCD147 with CAS was more pronounced in males, smokers, and participants with T2DM or dyslipidemia than in females, nonsmokers, and participants without T2DM or dyslipidemia. A study indicates that the incidence of premature ASCVD is significantly higher for men aged < 50 years than for women aged < 55 years (46–53 vs. 18–23 per 100, 000) [57]. This sex-related difference gradually declines but remains significant, even though estrogen levels decrease in postmenopausal women [58]. Smoking significantly increases the risk of ASCVD, and a longer period of smoking cessation is associated with a lower risk of ASCVD [59]. Diabetes accelerates the process of atherosclerosis by increasing low-density lipoprotein cholesterol and advanced glycation end products and activating pro-inflammatory signaling pathways [60]. Dyslipidemia leads to the accumulation of lipids within the arterial wall, promoting plaque formation. Specifically, elevated low-density lipoprotein cholesterol contributes to the formation of fatty streaks in arterial walls by infiltrating the endothelium and triggering inflammatory responses, while decreased high-density lipoprotein cholesterol impairs the body’s ability to clear excess cholesterol from the bloodstream, promoting plaque buildup [61].

Several limitations exist in the research. Firstly, the study was conducted in Chinese Han population. Whether the result can be generalized to other ethnicities remains to be further investigated. Secondly, the participants with a small sample size were collected from the single center. Therefore, a prospective, multicenter, large-scale study is imperative to validate this result and determine the causality between GlyCD147 and CAS in the future. Thirdly, the differential expression analysis of the GSE43292 dataset applied a standard two-group comparison workflow without accounting for patient pairing, which may introduce bias in variance estimation and significance testing. Further analysis using a linear model appropriate for paired designs to validate the differential expression results is warranted. Finally, MMP9 levels were not measured, and correlation or mediation analysis between GlyCD147 and MMP9 was not conducted due to limited clinical samples and budget constraints. While bioinformatics analysis determined MMP9 as the top hub gene in CAS and provided a hypothetic mechanistic link, the proposed mechanism of the GlyCD147-MMP9 inflammatory response pathway in CAS cannot be directly confirmed by the present observational design. Consequently, additional quantification of MMP9 and functional experiments remain to be performed to further elucidate the underlying pathological processes.

## Conclusion

This research, from three complementary perspectives, revealed that elevated serum GlyCD147 is independently associated with CAS. Building on this observational association and existing evidence, we propose a plausible hypothesis that GlyCD147 may contribute to CAS pathogenesis via an MMP9-mediated inflammatory response, which warrants further functional validation.

## Supplementary Information

Supplementary Material 1.

## Data Availability

The dataset generated and analyzed in the research are available from the corresponding authors on reasonable request.

## Abbreviations

ACTC1: Actin, alpha, cardiac muscle 1
ANPEP: Alanyl aminopeptidase, membrane
ASCVD: Atherosclerotic cardiovascular diseases
AUC: Area under the curve
BP: Biological processes
cAMP: Cyclic adenosine monophosphate
CAS: Carotid atherosclerosis
CC: Cellular component
CCR1: C-C motif chemokine receptor 1
CD147: Cluster of differentiation 147
CD163: Cluster of differentiation 163
CIMT: Carotid intima-media thickness
95% CI: 95% confidence intervals
CR1: Complement component 3b/4b receptor 1 (Knops blood group)
CXCL10: C-X-C motif chemokine ligand 10
DEG: Differentially expressed gene
ELISA: Enzyme-linked immune sorbent assay
EMMPRIN: Extracellular matrix metalloproteinase inducer
GCNT1: Core 2 β-1, 6-N-acetylglucosaminyltransferase-1
GEO: Gene expression omnibus
GlyCD147: Glycosylated CD147
GO: Gene Ontology
HECS: Health Examination Cohort Study
HG-CD147: Highly glycosylated CD147
IgG: Immunoglobulin G
ITGAX: Integrin subunit alpha X
KEGG: Kyoto Encyclopedia of Genes and Genomes
LG-CD147: Lowly glycosylated CD147
MF: Molecular function
MME: Membrane metallo-endopeptidase
MMP: Matrix metalloproteinases
OR: Odds ratio
Ox-LDL: Oxidized LDL
PLEK: Pleckstrin
PPI: Protein-protein interaction
PSM: Propensity score matching
ROC: Receiver operating characteristic
SD: Standard deviation
SMD: Standardized mean difference
STRING: Search tool for retrieval of interaction genes
ST3gal-IV: α-2, 3-sialyltransferase IV
SUMC: Shantou University of Medical College
T2DM: Type 2 diabetes mellitus
VSMC: Vascular smooth muscle cell
