# Supplementary material for: Association of GlyCD147 with carotid atherosclerosis: evidence from integrative analyses
Source: BMC Cardiovasc Disord. 2026 Feb 28;26:287. doi: 10.1186/s12872-026-05665-4 (PMC13059536; doi:10.1186/s12872-026-05665-4)
Supplement: Supplementary file 1 — Supplementary Material 1. [file 12872_2026_5665_MOESM1_ESM.docx]

**Association of GlyCD147 with Carotid Atherosclerosis:**

**Evidence from Integrative Analyses**

Cuihong Tian^1,2,3,4,5,6^, Peixuan Yang^7^, Xingang Li^6^, Hongxia Zhang^7^, Jieyi He^7^, Jinxiu Zhu^1,2,3,8,9^*, Yequn Chen^1,2,3^*, Xuerui Tan^1,2,3^*

1. Department of Cardiology, First Affiliated Hospital of Shantou University Medical College, Shantou, Guangdong 515041, China
2. Clinical Medical Research Centre, First Affiliated Hospital of Shantou University Medical College, Shantou, Guangdong 515041, China
3. Human Phenome Institute of Shantou University Medical College, Guangdong Engineering Research Centre of Human Phenome, Chemistry and Chemical Engineering Guangdong Laboratory, Shantou, Guangdong 515063, China
4. Glycome Research Institute, Shantou University Medical College, Shantou, Guangdong 515041, China
5. Molecular Cardiology Laboratory, First Affiliated Hospital of Shantou University Medical College, Shantou, Guangdong 515041, China
6. Centre for Precision Health, Edith Cowan University, Perth, WA 6027, Australia
7. Health Care Centre, First Affiliated Hospital of Shantou University Medical College, Shantou, Guangdong 515041, China

***Correspondence author**

**Contents**

[Methods for bioinformatic analysis 3](#_Toc28887)

[DEGs screening 3](#_Toc26133)

[GO and KEGG enrichment analyses 3](#_Toc31626)

[Construction of PPI network 3](#_Toc9096)

[Selection of hub genes 4](#_Toc23211)

[Reference 4](#_Toc12129)

[Table S1. Basic characteristics in the clinical case-control study. 5](#_Toc27794)

[Fig. S1. Propensity score distribution before (left) and after (right) PSM. 7](#_Toc17908)

[Fig. S2. Analysis of DEGs 7](#_Toc32290)

[Table S2. Top ten up- and down-regulated DEGs of GSE43292. 8](#_Toc25356)

[Table S3. GO and KEGG pathway enrichment analysis of DEGs. 9](#_Toc15520)

[Fig. S3. PPI network with 94 nodes and 110 edges. 11](#_Toc17627)

**Methods for bioinformatic analysis**

**DEGs screening**

GEO2R (<http://www.ncbi.nlm.nih.gov/geo/geo2r>), an online tool used for the identification of differentially expressed genes (DEGs) in two or more groups of samples, was applied to screen the DEGs between carotid plaques (stage IV and over of the Stary classification) and their adjacent tissues (stages I and II of the Stary classification). Statistically significant genes were determined by adjusted *P*-values. The Benjamini and Hochberg (BH) false discovery rate (FDR) was used for multiple testing corrections. Probe sets without gene titles or gene symbols were deleted, and genes with more than one probe set were averaged. Standard with adjusted *P*-value <0.05 and |log_2_ fold change (FC)|>1 was considered as the threshold for screening the statistically significant DEGs.

**GO and KEGG enrichment analyses**

The database for annotation, visualization and integrated discovery (DAVID, https://david.ncifcrf.gov/) was performed to analyze the function of DEGs. Gene Ontology (GO) enrichment analysis was used to classify the target genes into three categories: biological processes (BP), cellular component (CC) and molecular function (MF), based on the significance analysis, misjudgment rate analysis and enrichment degree analysis of discrete distribution. A *P*-value <0.05 denoted statistical significance. The metabolic pathways of DEGs were analyzed and explored by Kyoto Encyclopedia of Genes and Genomes (KEGG) to systematically evaluate the regulatory pathways of genes.

**Construction of PPI network**

The search tool for retrieval of interaction genes (STRING) (STRING, <http://string-db.org>) was applied for functional enrichment analysis of protein-protein interaction (PPI) networks. The PPI with a composite score >0.4 was regarded as statistical significance.

**Selection of hub genes**

Cytoscape [1] (<https://cytoscape.org/>), an open platform, was used for visualizing molecular interaction networks. The top ten hub genes with high degrees were evaluated and screened by the CytoHubba [2] Plugin in Cytoscape.

**Reference**

1. Otasek D, Morris JH, Bouças J, Pico AR, Demchak B: **Cytoscape Automation: empowering workflow-based network analysis**. *Genome Biol* 2019, **20**(1):185.

2. Chin CH, Chen SH, Wu HH, Ho CW, Ko MT, Lin CY: **cytoHubba: identifying hub objects and sub-networks from complex interactome**. *BMC Syst Biol* 2014, **8 Suppl 4**(Suppl 4):S11.

**Table S1. Basic characteristics in the clinical case-control study.**

|  | Before PSM | | | | After PSM | | | |
| --- | --- | --- | --- | --- | --- | --- | --- | --- |
| Variables | CAS  (n=124) | Controls  (n=100) | *P*-value | SMD | CAS  (n=69) | Controls  (n=69) | *P*-value | SMD |
| Female | 29 (23.4%) | 24 (24.0%) | >0.999 | 0.014 | 17 (24.6%) | 17 (24.6%) | >0.999 | <0.001 |
| Age, y | 57.6±4.9 | 55.4±3.9 | <0.001 | 0.508 | 57.2±5.2 | 56.3±4.1 | 0.168 | 0.182 |
| Smoking, % | 12 (9.7%) | 4 (4.0%) | 0.168 | 0.226 | 5 (7.2%) | 4 (5.8%) | >0.999 | 0.059 |
| Alcohol consumption, % | 12 (9.7%) | 4 (4.0%) | 0.168 | 0.226 | 5 (7.2%) | 4 (5.8%) | >0.999 | 0.059 |
| Hypertension, % | 35 (28.2%) | 20 (20.0%) | 0.206 | 0.193 | 16 (23.2%) | 17 (24.6%) | >0.999 | 0.034 |
| T2DM, % | 49 (39.5%) | 19 (19.0%) | 0.002 | 0.463 | 19 (27.5%) | 19 (27.5%) | >0.999 | <0.001 |
| Dyslipidemia, % | 57 (46.0%) | 19 (19.0%) | <0.001 | 0.601 | 15 (21.7%) | 19 (27.5%) | 0.553 | 0.135 |
| Obesity, % | 10 (8.1%) | 6 (6.0%) | 0.737 | 0.081 | 4 (5.8%) | 5 (7.2%) | >0.999 | 0.059 |
| BMI, kg/m^2^ | 24.50(23.10,26.05) | 24.50(22.90,25.25) | 0.244 | 0.163 | 24.50(22.85,25.85) | 24.50(22.85,25.30) | 0.623 | 0.075 |
| TG, mmol/L | 1.32(1.02,2.04) | 1.15(0.90,1.50) | 0.003 | 0.570 | 1.22(1.00,1.57) | 1.23(0.94,1.57) | 0.703 | 0.193 |
| TC, mmol/L | 5.46±1.09 | 5.07±0.72 | 0.002 | 0.420 | 5.26±0.88 | 5.17±0.75 | 0.945 | 0.107 |
| HDL-C, mmol/L | 1.33±0.36 | 1.40±0.30 | 0.127 | 0.208 | 1.39±0.34 | 1.38±0.33 | 0.775 | 0.016 |
| LDL-C, mmol/L | 3.33±0.91 | 3.08±0.65 | 0.023 | 0.302 | 3.21±0.73 | 3.17±0.69 | 0.995 | 0.063 |
| FBG, mmol/L | 5.88(5.51,6.53) | 5.51(5.14,5.98) | <0.001 | 0.519 | 5.78(5.32,6.17) | 5.64(5.19,6.16) | 0.182 | 0.134 |
| SBP, mmHg | 131(129,140) | 131(121,136) | 0.006 | 0.411 | 131(130,137) | 131(123,137) | 0.245 | 0.165 |
| DBP, mmHg | 85(81,92) | 85(79,90) | 0.155 | 0.201 | 85(53,89) | 85(80,90) | 0.552 | 0.083 |
| CD147, μg/L | 10.50(9.78,11.46) | 10.80(9.95,11.62) | 0.128 | 0.214 | 10.49(9.83,11.46) | 10.65(9.73,11.62) | 0.574 | 0.201 |
| GlyCD147, μg/L | 2.56(1.95,3.57) | 1.19(0.81,1.78) | <0.001 | 0.954 | 2.40(1.48,3.96) | 1.06(0.77,1.78) | <0.001 | 0.973 |

Abbreviations: BMI: body mass index; CAS: carotid atherosclerosis; CD147: cluster of differentiation 147; DBP: diastolic blood pressure; FBG: fasting blood glucose; GlyCD147: Glycosylated CD147; HDL-C: high-density lipoprotein cholesterol; LDL-C: low-density lipoprotein cholesterol; PSM:propensity score matching; SBP: systolic blood pressure; SMD: standardized mean difference; TC: total cholesterol; TG: triglyceride; T2DM: type 2 diabetes mellitus.

**
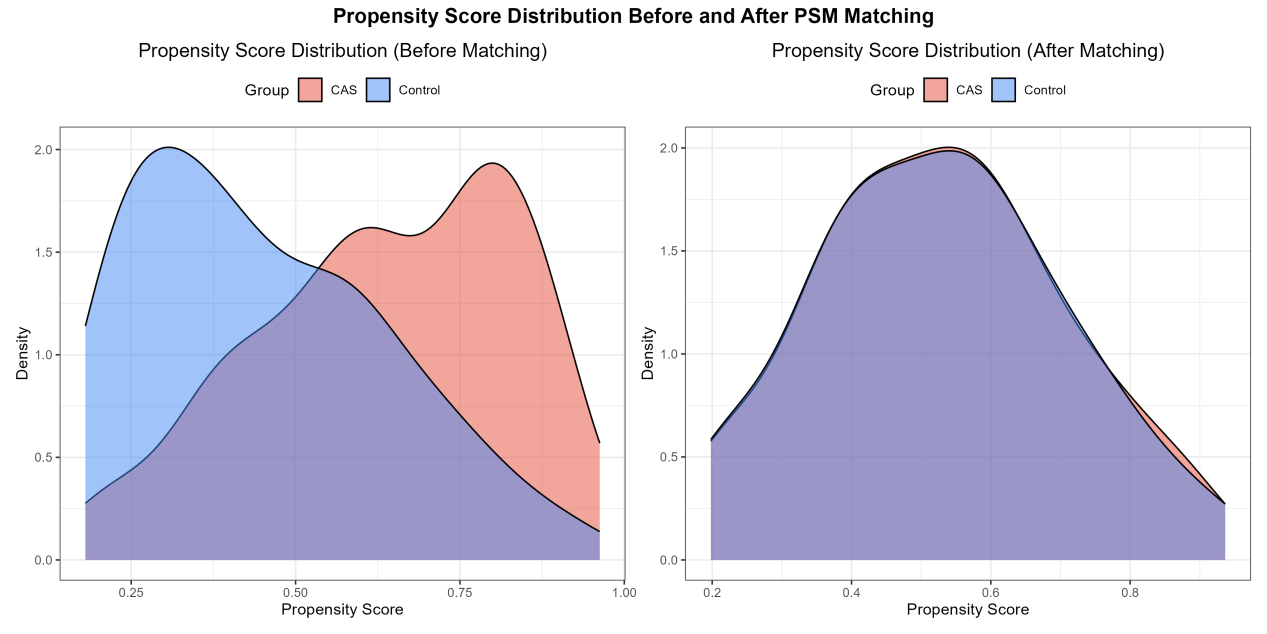
**

**Fig. S1. Propensity score distribution before (left) and after (right) PSM.** Covariates including gender, age, smoking history, alcohol consumption history, hypertension, T2DM, dyslipidemia, and obesity were matched by PSM. Abbreviation: PSM: propensity score matching.

**
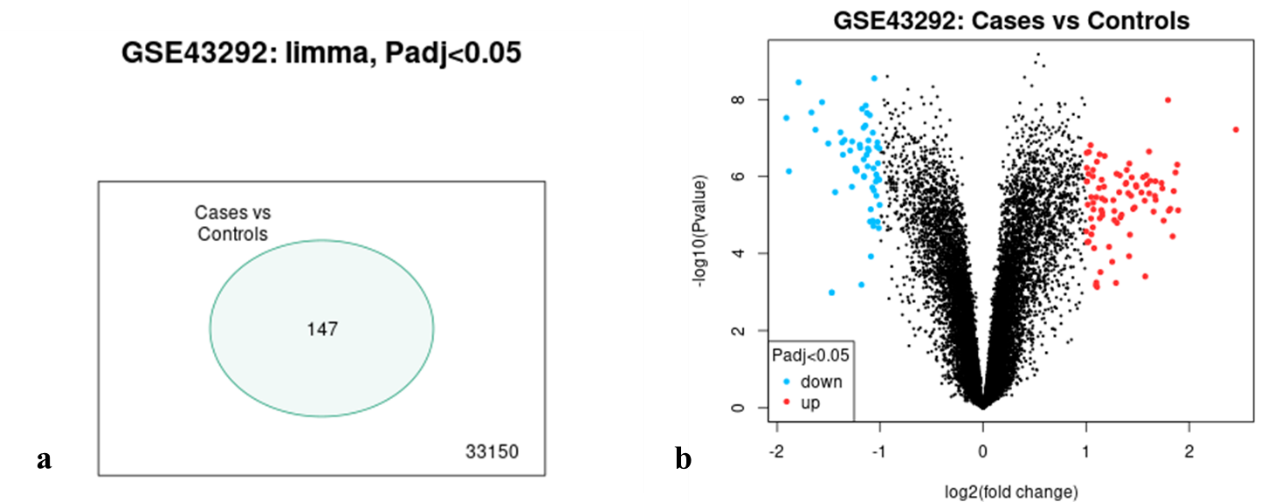
**

**Fig. S2. Analysis of DEGs.** a. Venn diagram; b. Volcano map. Screening parameters were set at |log_2_FC|>1 and adjusted P-value <0.05. Red dots represent upregulated genes, blue dots represent downregulated genes, genes with no significant changes are labeled as black dots. Cases and controls refer to carotid plaques (stage IV and over of the Stary classification) and their adjacent tissues (stages I and II of the Stary classification), respectively.

**Table S2. Top ten up- and down-regulated DEGs of GSE43292.**

| Upregulated genes | | | Downregulated genes | | |
| --- | --- | --- | --- | --- | --- |
| Gene symbol | adj. *P*-value | Log_2_FC | Gene symbol | adj. *P*-value | Log_2_FC |
| FABP4 | 6.05E-08 | 2.454461 | CNTN1 | 3.00E-08 | -1.91103 |
| JCHAIN | 7.54E-06 | 1.893149 | TPH1 | 7.30E-07 | -1.88663 |
| MMP7 | 3.57E-05 | 1.840231 | CNTN4 | 3.57E-09 | -1.79233 |
| MMP9 | 6.88E-06 | 1.817804 | CASQ2 | 2.16E-08 | -1.66766 |
| CD36 | 7.71E-06 | 1.802205 | MYOCD | 6.08E-08 | -1.62845 |
| IBSP | 1.03E-08 | 1.794982 | FHL5 | 1.17E-08 | -1.56465 |
| DPP4 | 2.24E-07 | 1.610873 | CARTPT | 1.03E-03 | -1.47049 |
| MMP12 | 3.92E-04 | 1.574106 | ATRNL1 | 2.54E-06 | -1.43750 |
| MME | 1.67E-06 | 1.485000 | RPS6KA6 | 1.30E-07 | -1.36920 |
| ATP6V0D2 | 3.24E-05 | 1.425070 | CNTN3 | 2.71E-07 | -1.36174 |

Abbreviations: ATP6V0D2: ATPase H+ transporting V0 subunit d2, ATRNL1: attractin like 1, CARTPT: cocaine- and amphetamine-regulated transcript prepropeptide, CASQ2: calsequestrin 2, CD36: cluster of differentiation 36, CNTN1: contactin 1, CNTN3: contactin 3, CNTN4: contactin 4, DPP4: dipeptidyl peptidase 4, FABP4: fatty acid binding protein 4, FHL5: four and a half LIM domains 5, IBSP: integrin binding sialoprotein, JCHAIN: joining chain of multimeric immunoglobulin A and immunoglobulin M, MME: membrane metallo endopeptidase, MMP7: matrix metallopeptidase 7, MMP9: matrix metallopeptidase 9, MMP12: matrix metallopeptidase 12, MYOCD: myocardin, RPS6KA6: ribosomal protein S6 kinase A6, TPH1: tryptophan hydroxylase 1.

**Table S3. GO and KEGG pathway enrichment analysis of DEGs.**

| Category | Term | Description | Counts | *P*-value |
| --- | --- | --- | --- | --- |
| BP term | GO:0006955 | Immune response | 11 | 8.44E-05 |
| BP term | GO:0007155 | Cell adhesion | 10 | 7.84E-04 |
| BP term | GO:0006508 | Proteolysis | 10 | 0.001426 |
| BP term | GO:0007399 | Nervous system development | 6 | 0.019058 |
| BP term | GO:0045087 | Innate immune response | 7 | 0.027975 |
| CC term | GO:0005615 | Extracellular space | 21 | 1.41E-05 |
| CC term | GO:0005576 | Extracellular region | 21 | 1.73E-04 |
| CC term | GO:0070062 | Extracellular exosome | 28 | 7.39E-04 |
| CC term | GO:0005887 | Integral component of plasma membrane | 18 | 8.39E-04 |
| CC term | GO:0005886 | Plasma membrane | 33 | 0.007421 |
| MF term | GO:0004222 | Metalloendopeptidase activity | 6 | 2.49E-04 |
| MF term | GO:0030246 | Carbohydrate binding | 7 | 4.35E-04 |
| MF term | GO:0004252 | Serine-type endopeptidase activity | 7 | 0.001703 |
| MF term | GO:0005509 | Calcium ion binding | 9 | 0.026098 |
| MF term | GO:0004175 | Endopeptidase activity | 3 | 0.029625 |
| KEGG pathway | hsa00380 | Tryptophan metabolism | 4 | 0.001666 |
| KEGG pathway | hsa04024 | cAMP signaling pathway | 6 | 0.006064 |
| KEGG pathway | hsa04720 | Long-term potentiation | 4 | 0.006934 |
| KEGG pathway | hsa04640 | Hematopoietic cell lineage | 4 | 0.014736 |
| KEGG pathway | hsa04713 | Circadian entrainment | 4 | 0.018624 |

Abbreviations: BP: biological processes, cAMP: cyclic adenosine monophosphate, CC: cellular component, KEGG: kyoto encyclopedia of genes and genomes, MF: molecular function.


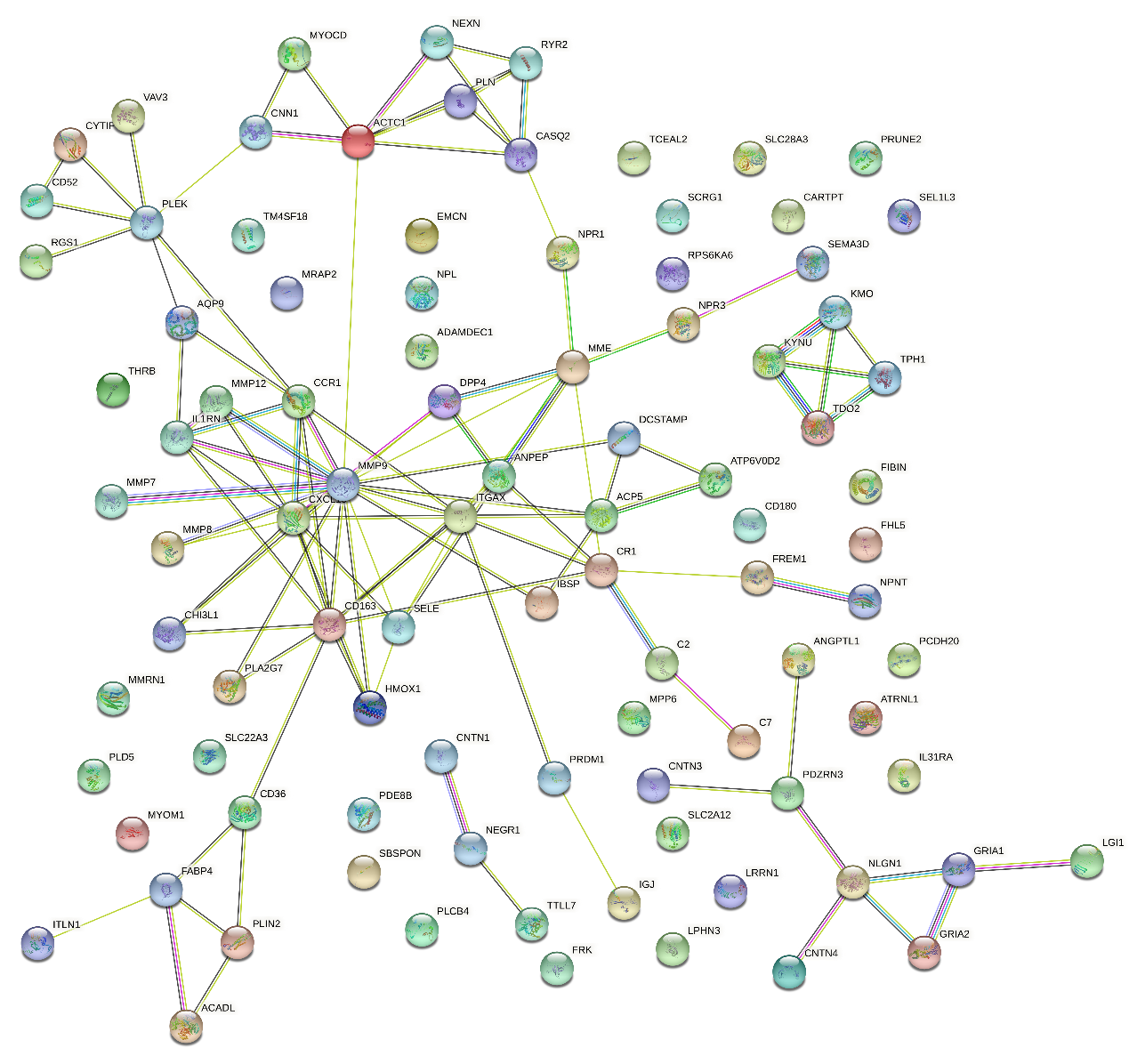


**Fig. S3. PPI network with 94 nodes and 110 edges.**
